# Severe anaphylactic shock reaction upon rituximab rechallenge in membranous nephropathy: a case report and literature review

**DOI:** 10.3389/fphar.2026.1788362

**Published:** 2026-05-29

**Authors:** Weidong Huang, Yuanyuan Xu, XiaoXiao Jin, Qiankun Zhang, Tingyan Xiang, Wenhui Lei

**Affiliations:** 1 Department of Nephrology, The Fifth Affiliated Hospital of Wenzhou Medical University, Lishui, Zhejiang, China; 2 Department of Pathology,The Fifth Affiliated Hospital of Wenzhou Medical University, Lishui, Zhejiang, China; 3 Department of Medicine, Liandu District Baiyun Street Community Health Service Center, Lishui, Zhejiang, China; 4 Department of Rheumatology, The Fifth Affiliated Hospital of Wenzhou Medical University, Lishui, Zhejiang, China

**Keywords:** adverse drug reaction, case report, hypersensitivity reaction, membranous nephropathy, rituximab

## Abstract

**Background:**

Rituximab, an anti-CD20 monoclonal antibody, is an established treatment for primary membranous nephropathy (PMN). While generally well-tolerated, its recognized adverse effects include infusion-related reactions and immediate hypersensitivity. Particularly severe manifestations occurring >24 h post-infusion remain rarely reported.

**Case Presentation:**

A 52-year-old male with a 7-year history of biopsy-proven PMN achieved complete remission after two uneventful courses of rituximab (1 g each) in 2018. He presented in August 2025 with nephrotic syndrome relapse. A third 1 g rituximab infusion was administered without immediate complications. Approximately 24 h post-infusion, he developed generalized urticaria, fever (38 °C), dysphagia, and arthralgia, progressing to hypotension (95/55 mmHg). Examination revealed diffuse, evanescent wheals. Laboratory findings showed elevated C-reactive protein, eosinophilia, and immunoglobulin E. Esophageal CT indicated localized wall edema, while infection was excluded. A diagnosis of severe hypersensitivity reaction (anaphylactic shock, acute urticaria, and angioedema) was made, consistent with Brown grade IV severity. He responded rapidly to intravenous methylprednisolone, antihistamines, and supportive care.

**Conclusion:**

This report highlights the potential for life-threatening hypersensitivity upon rituximab re-challenge, even with a remote history of uneventful use. It emphasizes the need for prolonged post-infusion observation, patient education regarding delayed symptoms, and preparedness for immediate management of severe allergic phenomena. Further studies are warranted to elucidate risk factors and mechanisms underlying these rare but serious events.

## Introduction

Membranous nephropathy (MN) represents a leading cause of nephrotic syndrome in adults, with approximately 75% of cases classified as idiopathic or primary MN (PMN), an autoimmune disorder primarily driven by antibodies against podocyte antigens (Ponticelli, 2025). The remaining cases are secondary to various etiologies, including autoimmune diseases, infections, medications, and malignancies (Lai et al., 2015). Rituximab, a chimeric monoclonal antibody targeting the CD20 antigen expressed on B-lymphocytes, induces B-cell depletion through several mechanisms, thereby reducing pathogenic autoantibody production (Bondza et al., 2020). Given the central role of B-cell dysfunction in MN pathogenesis, rituximab has emerged as a cornerstone of therapy for this condition.

With expanding clinical use, reports of rituximab-associated adverse events have accumulated. Commonly documented reactions include infusion-related reactions (IRRs) (Paul and Cartron, 2019), hypersensitivity responses (Fouda and Bavbek, 2020), increased infection risk (Lemoine et al., 2023), and cardiovascular events (Sharif et al., 2017). Overall, rituximab remains well-tolerated, with a relatively low incidence of severe adverse events. (Spinner et al., 2015). This article presents a case of severe hypersensitivity following rituximab administration in a patient with MN. We aim to contribute to the understanding of rituximab’s safety profile in clinical practice, providing insights to support its rational and secure therapeutic application.

## Case Presentation

**FIGURE 1:**
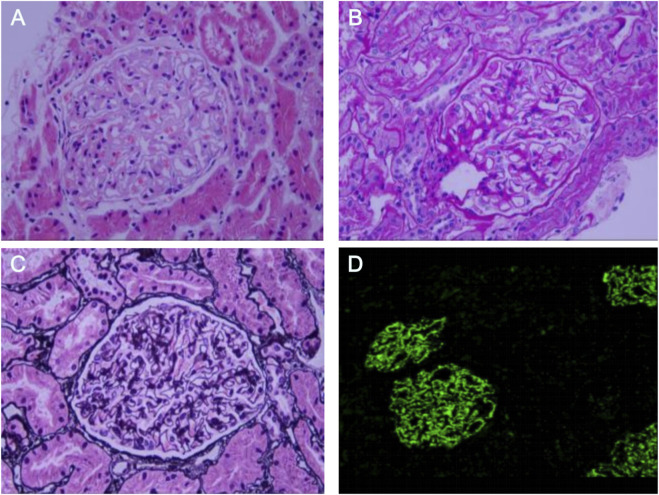
Renal biopsy pathology findings at initial diagnosis (2018). **(A)** Hematoxylin and eosin (H&E) stain at ×400 magnification demonstrates glomerular capillary loops with a rigid appearance. **(B)** Periodic acid-Schiff (PAS) stain at ×400 magnification reveals mild mesangial hypercellularity and matrix expansion. **(C)** Periodic acid-silver methenamine (PASM) stain at ×400 magnification shows diffuse glomerular basement membrane thickening with occasional spike-like projections. **(D)** Immunofluorescence microscopy for IgG at ×400 magnification reveals granular deposits along the glomerular capillary walls in a diffuse, global pattern.

A 52-year-old Asian male initially presented to our hospital in 2018 with lower extremity edema and massive proteinuria. A renal biopsy confirmed a diagnosis of stage II membranous nephropathy (Figure 1). Initial treatment consisted of a full course of corticosteroids combined with cyclosporine, followed by corticosteroid therapy with intravenous cyclophosphamide, both of which yielded suboptimal responses. Treatment was subsequently switched to rituximab. The patient received two intravenous doses of rituximab (1 g each), with both infusions completed uneventfully and no observed adverse drug reactions. Following this regimen, his nephropathy achieved complete remission. For the subsequent 7 years, he remained free of edema, and annual urinalysis showed no proteinuria. He had a history of hypertension but was not on antihypertensive medication, with blood pressure typically fluctuating between 130 and 160/80–100 mmHg.

In August 2025, the patient was re-admitted due to recurrent edema over the preceding 2 weeks. On admission (August 14), his blood pressure was 150/86 mmHg. Laboratory findings (Table 1) demonstrated marked proteinuria, hypoalbuminemia, and elevated serum anti–phospholipase A2 receptor (anti-PLA2R) IgG antibodies, consistent with disease relapse. Diuretic management with furosemide was initiated after admission. After excluding contraindications such as active infection and malignancy, and considering his prior treatment history, a dose of rituximab (1 g) was administered intravenously on August 18, starting at 11:30 and concluding at 21:20. Prior to rituximab infusion, the patient received prophylactic treatment with intravenous dexamethasone 5 mg and promethazine hydrochloride 12.5 mg. The infusion was well tolerated, with no immediate adverse reactions. Due to persistently elevated blood pressure readings during hospitalization, antihypertensive medication was advised but declined by the patient. He was discharged on August 19 without additional medications and advised to rest at home.

**TABLE 1:** Laboratory findings on admission.

The laboratory parameters	Inspection results on August 15th	Inspection results on August 23rd	Reference range
White blood cells	6.0	5.6	3.5–9.5 × 10^9^/L
Haemoglobin	147	163	130–175 g/L
Neutrophil percentage	60.4	67.4	40.0%–75.0%
Absolute eosinophil count	0.08	1.22	0.02–0.52 × 10^9^/L
Platelet	103	101	125–350 × 10^9^/L
ESR	25	30	0–15 mm/1h
CRP	6.21	56	<8 mg/L
Procalcitonin	0.02	0.04	<0.05 ng/mL
Albumin	30.6	25.0	40–55 g/L
Creatinine	78	104	57-111umol/L
24 h Urine protein	5.25	Not performed	<0.15g/24h
Anti-PLA2R IgG	78.16	Not performed	0-14RU/mL
ANA	Negative	Not performed	Negative
ANCA	Negative	Not performed	Negative
Anti GBM antibody	Negative	Not performed	Negative
T-SPOT.TB	Negative	Not performed	Negative
Absolute Th count	591	696	432–1341/μL
Absolute Tc cell count	243	334	238–1075/μL
Total T Cell count	849	1048	797–2370/μL
Total B Cell count	228	45	96–412/μL
IgG	4.72	4.79	7–16 g/L
IgE	6	1028	<100IU/mL
Complement C3	1.52	1.10	0.90–1.80 g/L
Complement C3	0.41	0.21	0.10–0.40 g/L
Interleukin-6	Not performed	15.71	0–7.13 pg/mL
Interleukin-10	Not performed	0.70	0–4.58 pg/mL
TNF-α	Not performed	0.92	0–3.23 pg/mL
TNF-β	Not performed	1.04	0–6.9 pg/mL

**FIGURE 2:**
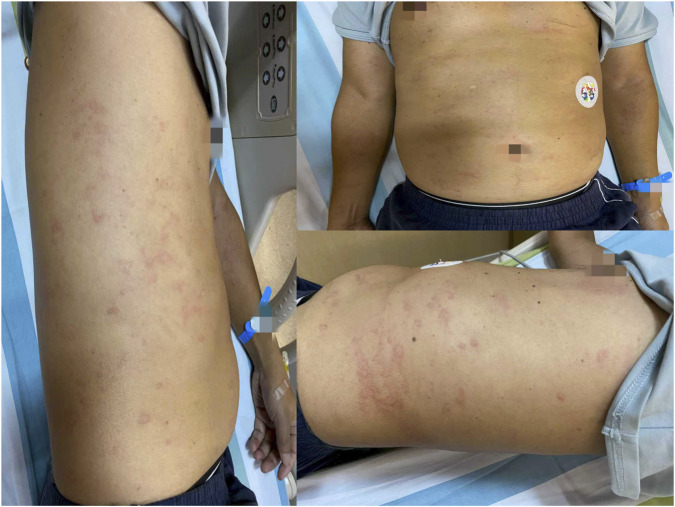
Clinical presentation of acute cutaneous hypersensitivity reaction. Diffuse, erythematous urticarial wheals are evident on the upper extremities and trunk. Some lesions appear confluent, forming larger plaques. The rash was evanescent, resolving without post-inflammatory changes.

**FIGURE 3:**
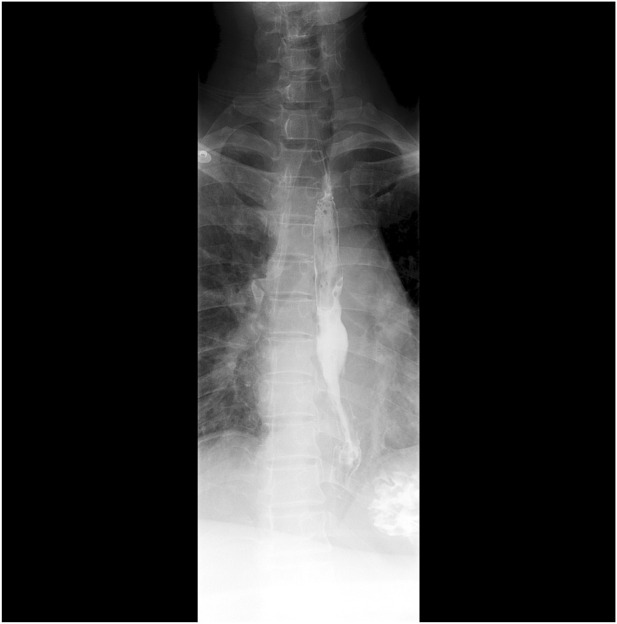
Esophagogram performed during the acute reaction (22 August 2025). No luminal narrowing, filling defects, or mucosal abnormalities are observed. The study was interpreted as normal, supporting a diagnosis of functional dysphagia secondary to angioedema rather than mechanical obstruction.

**FIGURE 4:**
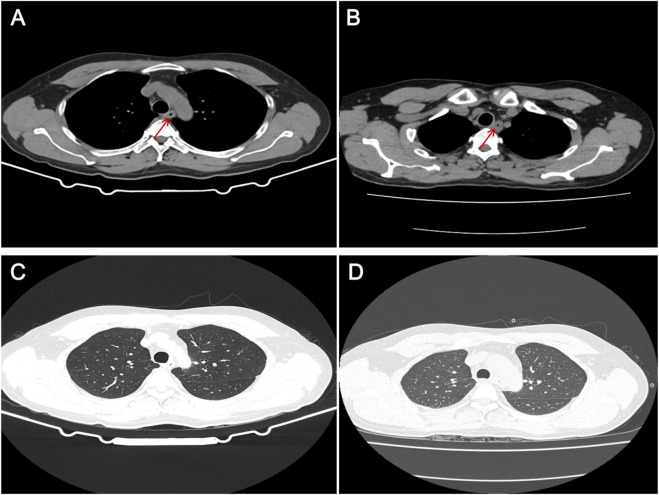
Serial thoracic computed tomography (CT) scans before and after rituximab rechallenge. **(A)** Mediastinal window (15 August 2025) shows normal esophageal wall thickness (red arrow). **(B)** Mediastinal window (22 August 2025) demonstrates focal esophageal wall thickening and edema with minimal peri-esophageal stranding (red arrow), consistent with angioedema. **(C)** Lung window (15 August 2025) shows clear lung fields without consolidation or ground-glass opacities. **(D)** Lung window (22 August 2025) remains clear, with no radiographic evidence of pulmonary infection or infiltrates. Comparison confirms the interval development of esophageal wall edema without new pulmonary findings.

On August 20, the patient developed a generalized pruritic rash at home without clear precipitating factors; the rash was transient and self-resolving. By August 21, he developed fever (maximum temperature 38 °C), intermittent dysphagia, coughing on swallowing, fatigue, and bilateral knee pain severe enough to limit ambulation. No chills, rigors, cough, or sputum production were reported. He returned to our hospital for admission on August 22. Physical examination revealed hypotension (95/55 mmHg) and widespread erythematous wheals, some confluent, which resolved without residual marks (Figure 2). Laboratory results (Table 1) demonstrated elevated C-reactive protein (CRP), eosinophil count, and immunoglobulin E (IgE) levels. A barium esophagogram was normal (Figure 3), while chest and esophageal computed tomography (CT) revealed localized edema and thickening of the esophageal wall without evidence of active pulmonary infection (Figure 4). Based on dermatological consultation and the clinical presentation of hypotension (95/55 mmHg, representing a significant decrease from baseline), diffuse urticaria, and associated systemic symptoms, a diagnosis of anaphylactic shock-most likely drug-related—was established. Therapeutic management included intravenous methylprednisolone at 80 mg daily for 3 days, tapered to 40 mg daily for an additional 4 days, and then discontinued. Calcium gluconate was administered at 2 g daily for 5 days. Fexofenadine hydrochloride was given at 180 mg daily for 7 days. Intravenous fluids consisted of normal saline at 1,500 mL daily for 3 days. Supportive care comprised enteral nutrition solution at 500 mL daily, omeprazole 40 mg daily, and hydrotalcite 1.5 g daily, each administered for 7 days. After 1 week of treatment, the rash resolved, fever subsided, and symptoms of dysphagia and coughing on swallowing abated. Follow-up laboratory parameters showed significant improvement.

## Discussion

This report describes a 52-year-old male patient with a 7-year history of membranous nephropathy (MN). He previously received rituximab treatment with a favorable response and no adverse events during initial administration. However, following re-administration of rituximab for disease relapse, he developed a severe allergic reaction approximately 24 h after infusion completion, characterized by anaphylactic shock, acute urticaria, and angioedema. The patient’s condition improved following aggressive management with glucocorticoids and other anti-allergic therapies. According to the Brown grading system (Brown, 2004), this reaction was classified as severe. Key points from this case include (Ponticelli, 2025): Close monitoring and follow-up are warranted for allergic reactions occurring beyond 24 h post-rituximab infusion (Lai et al., 2015); Severe adverse reactions can occur upon re-administration even if the initial course was well-tolerated; and (Bondza et al., 2020) Clinical vigilance should extend beyond cutaneous manifestations to include potential involvement of the gastrointestinal, respiratory, and other organ systems.

Currently, several classification systems exist for adverse reactions to biological agents like rituximab. In 2006, Pichler proposed a novel classification based on the unique characteristics of biologics, comprising five categories: type α (cytokine release syndrome); type β (hypersensitivity reactions, including IgE-, IgG-, and T-cell-mediated); type γ (immune dysregulation syndromes); type δ (cross-reactivity reactions); and type ε (non-immunological adverse effects) (Pichler, 2006). This framework aids in understanding clinical phenotypes, identifying risk factors, and guiding research. In 2018, Ghislain et al. proposed another classification. This system delineates four reaction patterns: Type I (IgE/non-IgE mediated), cytokine release reactions, mixed reactions (Type I/cytokine release), and delayed Type IV reactions (Isabwe et al., 2018).

The immunopathogenesis of rituximab-associated hypersensitivity is complex. Type I reactions are primarily IgE-mediated, where drug components act as allergens, inducing IgE production. Upon re-exposure, IgE cross-links on mast cells and basophils, triggering degranulation and release of histamine and other mediators (Khan, 2016). Type II reactions may involve antibody-dependent cellular cytotoxicity (ADCC) or complement activation, leading to target cell destruction and tissue injury (Patel et al., 2017). Type III reactions result from immune complex formation and deposition; rituximab as a foreign protein may stimulate antibody production, forming complexes that deposit in tissues, activate complement, and initiate inflammatory cascades, manifesting as serum sickness (Nakamura et al., 2020). Type IV reactions are T-cell-mediated, involving drug antigen presentation to T cells, subsequent cytokine release, and delayed inflammatory tissue damage (Fouda and Bavbek, 2020).

Applying these classifications, we discuss the potential mechanisms underlying this patient’s reaction. Infusion-related reactions (IRRs) are the most frequently reported adverse events associated with rituximab, typically occurring during the first infusion and presenting with flushing, fever, chills, and malaise (Smolders et al., 2025). These symptoms often respond to symptomatic measures or infusion rate reduction and usually do not recur (Bavbek et al., 2016). Cytokine release reactions are characterized by fever, nausea, pain, and rigors during the first infusion that do not respond to pre-medication or slower infusion rates. Symptoms are mediated by cytokine release, notably elevated serum TNF-α and IL-6 levels during the reaction (Isabwe et al., 2018). Type I reactions (IgE/non-IgE mediated) present with pruritus, urticaria, dyspnea, wheezing, hypotension, and potentially life-threatening anaphylaxis (Souqiyyeh et al., 2015), mediated by IgE or other immediate hypersensitivity mechanisms (Rajani et al., 2020). Mixed reactions combine features of cytokine release and IgE-mediated responses, with clinical manifestations including wheezing, flushing, urticaria, pruritus, fever and nausea. These may be associated with positive skin tests, rituximab-specific IgE, and elevated tryptase, IL-1, IL-6, or TNF-α (Patel and Khan, 2017). Serum sickness-like reactions (Type III hypersensitivity) are immune complex-mediated events, with symptoms such as fever, rash, and arthralgia typically appearing days to weeks after drug exposure (Bayer et al., 2019). Type IV (delayed) hypersensitivity reactions, often presenting as maculopapular eruptions, are less commonly reported but can range from mild exanthems to severe cutaneous adverse reactions (Fouda and Bavbek, 2020).

In the present case, the patient developed urticaria, fever, arthralgia, dysphagia, and coughing on swallowing approximately 24 h post-infusion. Examination revealed hypotension, diffuse erythematous wheals (partially confluent), and laboratory findings showed elevated CRP, eosinophils, and IgE. Esophageal CT indicated localized wall thickening and edema, while chest CT showed no evidence of active infection. After excluding acute infection, a diagnosis of anaphylactic shock, acute urticaria, and angioedema secondary to drug hypersensitivity was made. The predominance of urticaria and angioedema favors a Type I hypersensitivity mechanism driven by IgE, mast cells, and eosinophils. However, the ∼24-h delay, fever, arthralgia, and markedly elevated IL-6 suggest a concurrent cytokine-release process, while normal complement levels argue against a classic Type III mechanism. Thus, this case is best described as a mixed reaction: IgE-mediated Type I hypersensitivity as the primary driver of anaphylaxis and angioedema, with a substantial cytokine release component contributing to the delayed onset and systemic symptoms.

Diagnosis of rituximab hypersensitivity relies on clinical presentation, detailed history, and supportive investigations. A thorough history focusing on temporal relationship and symptom characteristics is crucial. Skin testing (prick or intradermal) can assess immediate hypersensitivity (Villarreal-González et al., 2021). Serological assays, such as measuring human anti-chimeric antibody (HACA) levels, may support diagnosis in serum sickness-like reactions (Nakamura et al., 2020). In this case, skin tests and drug-specific IgE assays were not performed, limiting definitive immunophenotyping. However, the temporal association, characteristic clinical findings, elevated eosinophils and IgE (previously normal), absence of concurrent infections or other culprit medications, and exclusion of alternative systemic causes strongly support rituximab as the trigger.

Most rituximab-induced hypersensitivity reactions are mild and manageable with drug discontinuation, infusion rate adjustment, or antihistamines. For severe reactions with respiratory distress or hypotension, immediate cessation of infusion, administration of intramuscular or subcutaneous epinephrine, intravenous corticosteroids, and fluid resuscitation are indicated (Souqiyyeh et al., 2015). This case, occurring 24 h post-infusion, underscores the need for extended clinical observation after rituximab administration—even in patients with prior uneventful exposures—to monitor for delayed reactions. Patient education regarding potential delayed adverse effects, including but not limited to rash, dysphagia, or systemic symptoms, is essential. For instance, progressive dysphagia and coughing on swallowing in this patient may reflect angioedema involving the oropharynx and upper esophagus, a recognized manifestation of severe hypersensitivity (Villarreal-González et al., 2021).

Although the patient had previously received rituximab without adverse events, the severe allergic reaction observed upon re-administration during disease relapse warrants consideration of factors beyond the drug itself. In the setting of hypoalbuminemia, rituximab pharmacokinetics may be substantially altered. In membranous nephropathy, rituximab has been associated with a shortened half-life, reduced systemic exposure, and increased clearance (Fogueri et al., 2018). Moreover, low albumin levels may elevate the free drug fraction, potentially increasing the risk of adverse reactions (Idasiak-Piechocka et al., 2025). Similar pharmacokinetic alterations have been observed with other highly protein-bound agents; for example, hypoalbuminemic patients receiving oral anticancer drugs with high protein binding are often less well tolerated, leading to early treatment discontinuation due to toxicity (Murdock et al., 2020). In membranous nephropathy, anti-PLA2R antibody titers closely track disease activity (Han et al., 2019). Although biologic therapies such as rituximab effectively reduce antibody levels and control disease activity, their use may also promote the formation of aberrant immune complexes and activate the complement system, potentially precipitating severe hypersensitivity reactions (Passerini et al., 2019; Manral et al., 2022). The chimeric (murine/human) structure of rituximab, particularly its murine-derived sequences, is implicated in immunogenicity and hypersensitivity. For patients who develop hypersensitivity to rituximab, alternative anti-CD20 monoclonal antibodies with different structural features, such as obinutuzumab or ofatumumab, may be considered as potentially better-tolerated alternatives (Vivarelli et al., 2017; Podestà et al., 2024).

This report has several limitations. First, as a single-center case report, the findings are based on one patient, limiting generalizability regarding the incidence or broader clinical spectrum of rituximab hypersensitivity. Second, although the clinical presentation—particularly urticaria, elevated IgE, and eosinophilia—strongly suggests an IgE-mediated (Type I) mechanism with a possible cytokine release component, the lack of skin testing or rituximab-specific IgE assays means definitive immunological classification remains presumptive. Third, while we hypothesize that hypoalbuminemia may have altered rituximab pharmacokinetics and contributed to reaction severity, no therapeutic drug monitoring was performed to confirm this. Fourth, although IL-6 elevation supports a cytokine release process, the absence of serial cytokine measurements limits our ability to temporally correlate these changes with clinical course. Finally, the mechanistic discussion is based on literature review and clinical inference; future studies incorporating drug-specific antibody testing, complement activation markers, and genetic predisposition factors are needed to elucidate the precise pathogenic mechanisms and identify at-risk patients.

## Conclusion

We report a case of severe hypersensitivity reaction following non-initial rituximab administration in a patient with membranous nephropathy. This case highlights several key clinical considerations (Ponticelli, 2025): Severe allergic reactions can occur upon re-administration of rituximab even when previous courses were well-tolerated, emphasizing that prior tolerance does not guarantee future safety (Lai et al., 2015). Hypersensitivity manifestations can involve multiple organ systems (e.g., cutaneous, gastrointestinal, musculoskeletal), necessitating comprehensive monitoring and evaluation beyond typical infusion-related symptoms (Bondza et al., 2020). Given the potential for delayed onset, extended clinical observation periods and thorough patient education regarding late-appearing symptoms are crucial following rituximab infusion (Paul and Cartron, 2019). Prompt recognition and management—including immediate drug discontinuation and aggressive anti-allergic therapy with glucocorticoids and supportive measures—are essential in severe cases.

This case adds to the growing literature on severe hypersensitivity reactions to biological therapies and underscores the need for continued vigilance in patients receiving rituximab, particularly upon re-exposure. Future prospective registries and mechanistic studies focusing on biomarkers of immunogenicity (e.g., HACA, drug-specific IgE/IgG) and host genetic factors are needed to identify at-risk patients and personalize monitoring strategies.

## Data Availability

The original contributions presented in the study are included in the article/supplementary material, further inquiries can be directed to the corresponding author.
